# A synthetic BBB-permeable tripeptide GCF confers neuroprotection by increasing glycine in the ischemic brain

**DOI:** 10.3389/fphar.2022.950376

**Published:** 2022-08-15

**Authors:** Juan Chen, Yang Zhuang, Ya Zhang, Huabao Liao, Rui Liu, Jing Cheng, Zhifeng Zhang, Jiangdong Sun, Jingchen Gao, Xiyuran Wang, Shujun Chen, Liang Zhang, Fengyuan Che, Qi Wan

**Affiliations:** ^1^ Department of Neurology, The Central Hospital of Wuhan, Tongji Medical College, Huazhong University of Science and Technology, Wuhan, China; ^2^ Department of Physiology, School of Medicine, Wuhan University, Wuhan, China; ^3^ Department of Pathophysiology, School of Basic Medicine, Institute of Neuroregeneration and Neurorehabilitation, Qingdao University, Qingdao, China; ^4^ Krembil Research Institute, University Health Network, University of Toronto, Toronto, ON, Canada; ^5^ Central Laboratory, Department of Neurology, Linyi People’s Hospital, Qingdao University, Linyi, China; ^6^ Qingdao Gui-Hong Intelligent Medical Technology Co., Ltd., Qingdao, China

**Keywords:** ischemic stroke, glycine, BBB, tripeptide, neuroprotection

## Abstract

**Background:** We and others have previously demonstrated that glycine is neuroprotective in cerebral ischemia-reperfusion injury. But glycine has low permeability to the blood–brain barrier (BBB). To deliver glycine into the ischemic brain to confer neuroprotection, we designed a novel glycine-containing and BBB-permeable tripeptide, the H-glycine-cysteine-phenylalanine-OH (GCF).

**Methods:** For the synthesis of GCF, phenylalanine was included to increase the BBB permeability of the tripeptide. Cysteine was conjugated with glycine to enable the release of glycine from GCF. With the use of immunofluorescence labeling and HPLC assays, we measured the distribution and level of GCF. We used TTC labeling, LDH release, and MTT assays to evaluate the neuroprotective effect of GCF.

**Results:** Following intravenous injection in a rat model of cerebral ischemia-reperfusion injury, GCF was intensively distributed in the ischemic neurons. Intravenous injection of GCF, but not the non-cleavable acetyl-GCF, resulted in the elevation of glycine in the ischemic brain. GCF but not acetyl-GC conferred neuroprotection in ischemic stroke animals.

**Conclusion:** GCF protects against cerebral ischemia-reperfusion injury in the rat. In contrast to peptide drugs that exert therapeutic effect by interfering with signaling interaction, GCF acts as a BBB shuttle and prodrug to deliver glycine to confer neuroprotection, representing a novel therapeutic strategy for acute ischemic stroke.

## Introduction

Glycine is the simplest non-essential amino acid that plays a fundamental role in cell metabolism (Perez-Torres et al., 2017; Razak et al., 2017). Glycine provides essential precursors for the synthesis of proteins, nucleic acids, and lipids that are crucial to cell growth and survival (Locasale, 2013). Glycine is an integral component of glutathione that is the main antioxidant molecule of the cell (Amelio et al., 2014). Glycine is also required to maintain the cellular redox balance (Petrat et al., 2012). In mitochondria, glycine fuels heme biosynthesis and sustains oxidative phosphorylation (Petrat et al., 2012).

In the central nervous system, glycine functions as an agonist of inhibitory glycine receptors and a co-agonist of excitatory NMDA receptors (Paul and de Belleroch e, 2014; Lynch et al., 2017), We and others have demonstrated that glycine exerted a neuroprotective effect *in vitro* and in cerebral ischemia injury *in vivo* (Yang et al., 2000; Zhao et al., 2005; Liu et al., 2007; Tanabe et al., 2010; Chen et al., 2016; Hu et al., 2016).

As the blood–brain barrier (BBB) prevents most drugs from reaching their targets in the brain, delivery of drugs into the brain is a major challenge in the drug development of neurological disorders. Peptides, acting as BBB shuttles, have recently received growing attention because of their lower cost, reduced immunogenicity, and higher chemical versatility, providing feasibility for the development of BBB-permeable CNS drugs (Oller-Salvia et al., 2016).

In this study, we showed that glycine had low BBB permeability in ischemic conditions. We, therefore, designed a glycine-containing and BBB-permeable tripeptide GCF. Intravenous injection of GCF led to the elevation of glycine in the ischemic neurons and protected against ischemic neuronal death in stroke animals.

## Results

### Glycine has low BBB permeability in cerebral ischemia conditions

**FIGURE 1:**
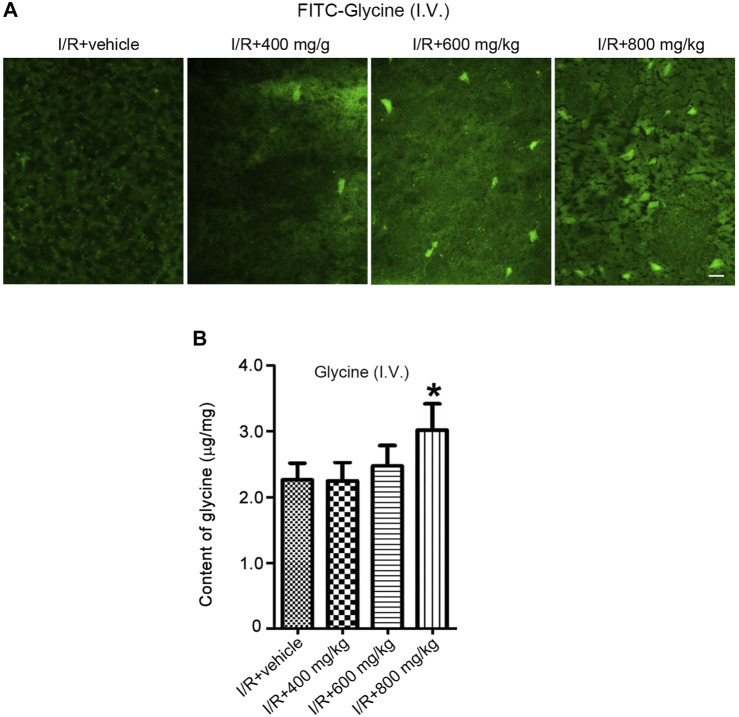
A high dose is required for glycine to pass through BBB after rat cerebral ischemia-reperfusion injury. **(A)** FITC-Glycine at different concentrations is intravenously injected at 1.0 h after rat cerebral I/R injury. Fluorescent images show the labeling of FITC-Glycine in the ischemia brain at 1.0 h after intravenous injection of FITC-Glycine. I/R: ischemia-reperfusion. Scale bar = 20 μm. **(B)** Glycine at different concentrations is intravenously injected at 1.0 h after rat cerebral I/R injury. The levels of glycine are measured by HPLC in the ischemic brain at 1.0 h after the intravenous injection of various concentrations of glycine (*n* = 8, *p < 0.05 vs. I/R + vehicle; ANOVA test). I/R: ischemia-reperfusion.

Glycine is known to protect against cerebral ischemia injury. However, the permeability of glycine to BBB is not clear. We set up to test whether glycine is permeable to BBB in rats subjected to middle cerebral artery occlusion (MCAO, an experimental model of cerebral ischemia injury). To enable the visualization of glycine in the brain, we conjugated fluorescein isothiocyanate (FITC) with glycine. FITC-Glycine was injected intravenously 1 h after cerebral ischemia-reperfusion injury in the MCAO rats. We found that only an extra high dose of FITC-Glycine (800 mg/kg) led to the significant detection of FITC-Glycine in the ipsilateral ischemic brain at 1.0 h after intravenous injection of FITC-Glycine (Figure 1A). To further confirm the low BBB permeability of glycine in the ischemic brain, we injected glycine intravenously 1 h after ischemia-reperfusion injury in the MCAO rats. HPLC assay showed that the levels of glycine were elevated in the ischemic brain at the concentration of 800 mg/kg after intravenous injection (Figure 1B). Thus, these results indicate that glycine has low BBB permeability in the ischemic brain and is unlikely to be directly used as a practical neuroprotectant for the treatment of stroke patients.

### Design a glycine-containing tripeptide that is BBB-permeable

**FIGURE 2:**
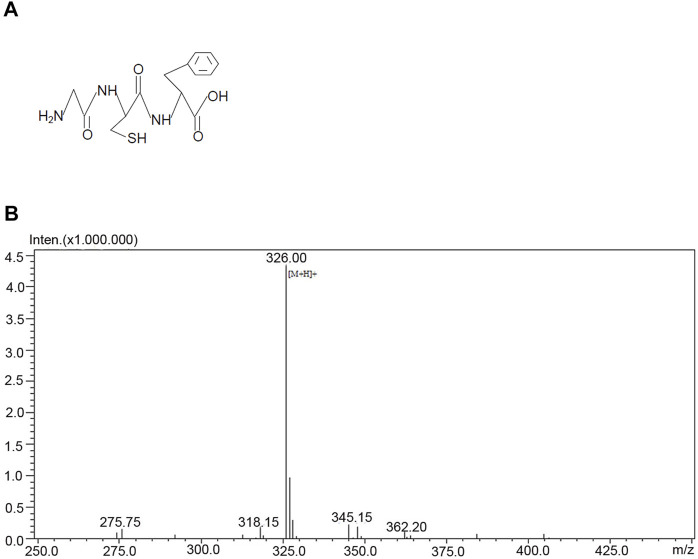
Structure and the mass spectrometry analysis of GCF. **(A)** The structure of GCF. **(B)** Mass spectrometry analysis of GCF.

To develop a practical approach that allowed glycine to pass through BBB and entered the injured brain to confer neuroprotection, we designed a glycine-containing tripeptide, the H-glycine-cysteine-phenylalanine-OH (GCF) (Figures 2A,B). As the aromatic amino acid phenylalanine has a high permeability to BBB (Rautio et al., 2013; Oller-Salvia et al., 2016), we included phenylalanine in the GCF to increase the permeability of GCF through BBB. We conjugated cysteine with glycine in the GCF as we reasoned that the bond between glycine and cysteine was cleavable.

**FIGURE 3:**
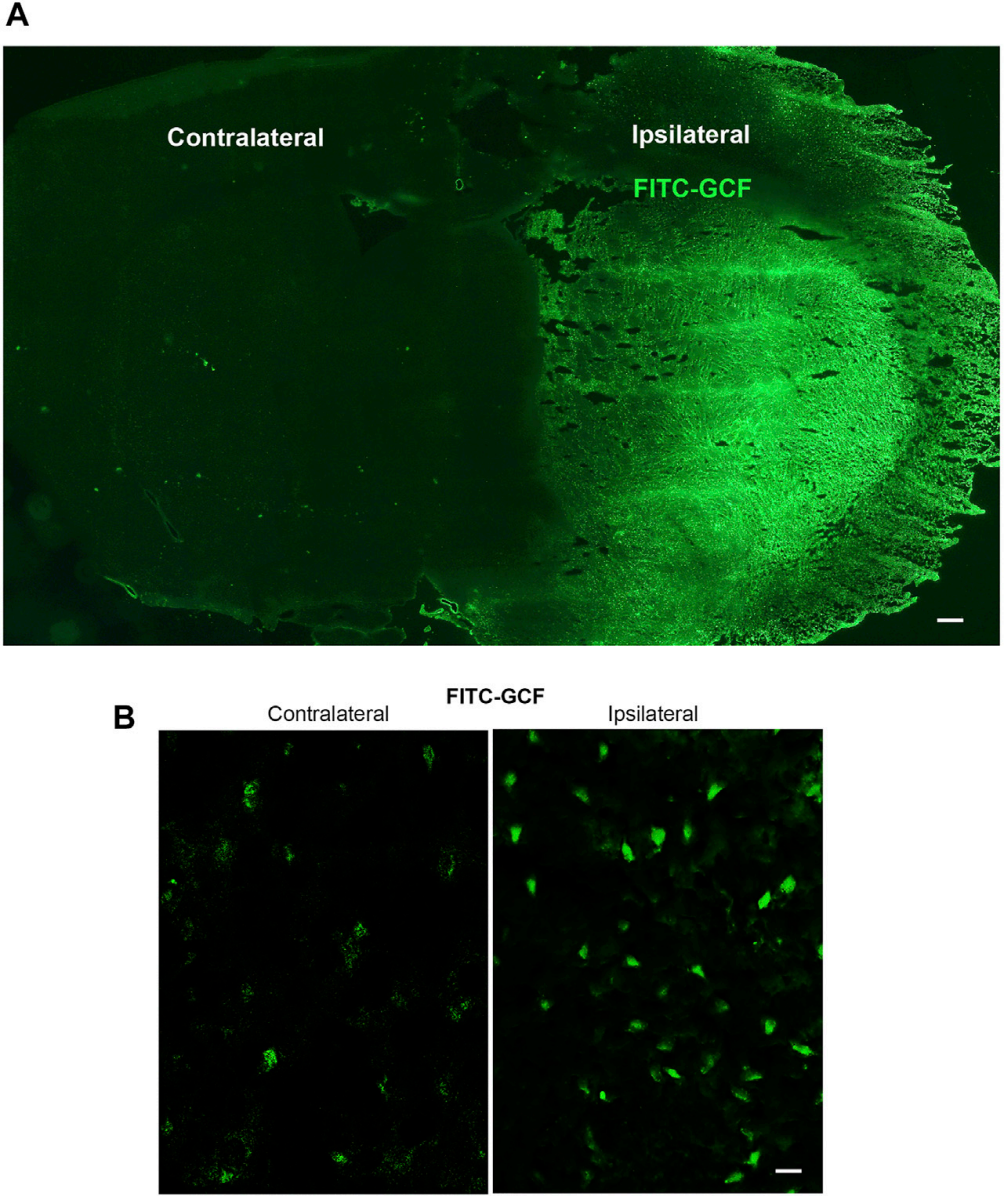
Intravenous injection of GCF leads to the distribution of GCF in the ischemic brain. **(A,B)** FITC-GCF (150 mg/kg) is intravenously injected at 1.0 h after rat cerebral ischemia-reperfusion injury. Brain sections are examined 1.0 h after intravenous FITC-GCF injection. **(A)** The low power sample image shows that FITC-GCF is distributed in the ipsilateral ischemic brain and the contralateral brain. Scale bar = 100 μm. **(B)** High-power images show the cell labeling by intravenous FITC-GCF injection in the ipsilateral and contralateral brain regions. Scale bar = 30 μm.

We first tested whether GCF was able to cross BBB and entered the ischemic brain in the rat MCAO model. To trace the location of GCF in the brain, we conjugated FITC with the amino terminal of GCF. FITC-GCF (150 mg/kg) was intravenously injected at 1.0 h after rat cerebral ischemia-reperfusion. At 1.0 h after intravenous injection, FITC-GCF was found to distribute both in the ipsilateral ischemic brain and the contralateral brain, but with high intensities in the ipsilateral brain (Figures 3A,B). These data suggest that GCF is permeable to rat BBB.

**FIGURE 4:**
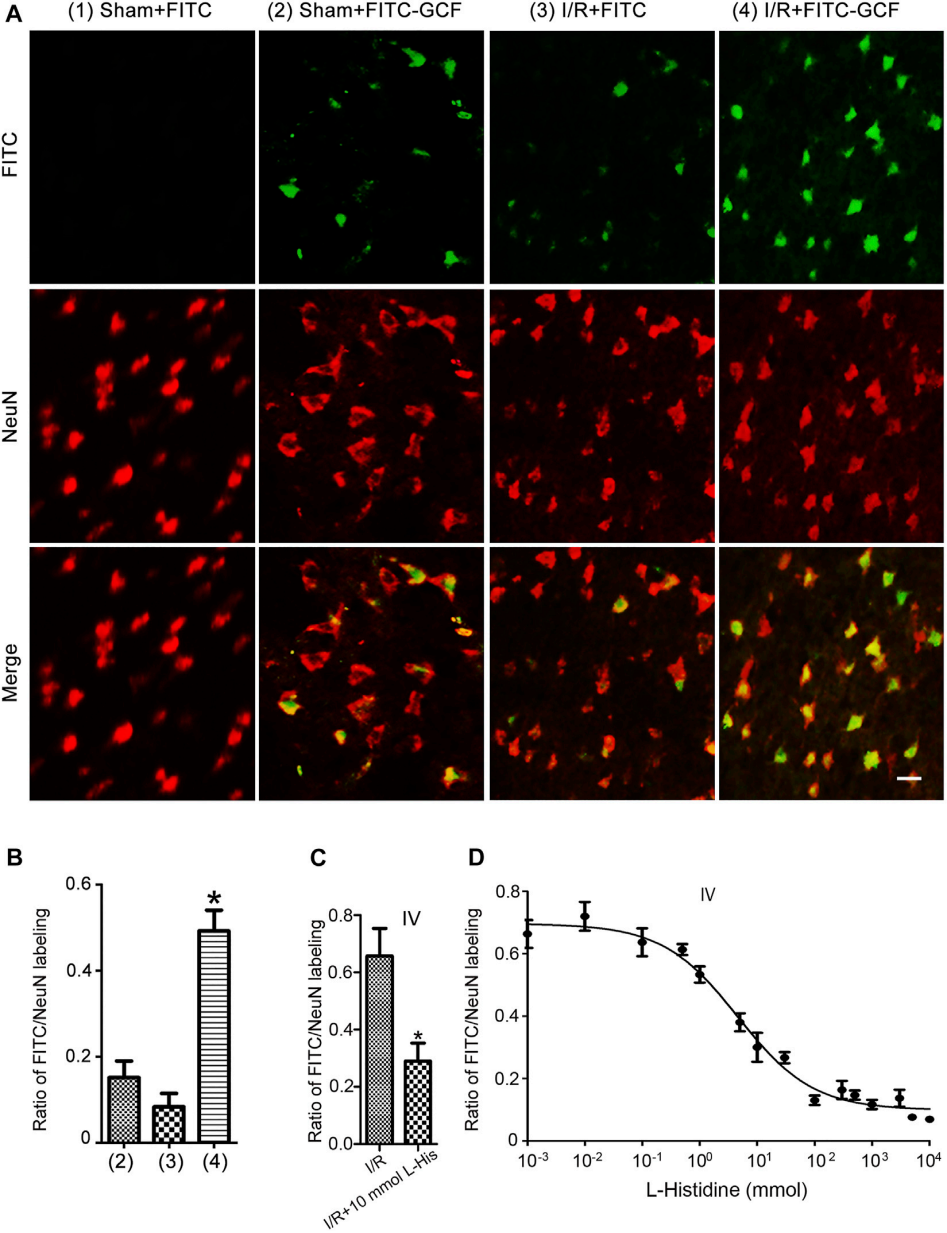
Intravenous injection of GCF leads to the distribution of GCF in the ischemic neurons. **(A)** Intravenous injection of FITC or FITC-GCF (150 mg/kg) is performed in rats at 1.0 h following I/R. The brain sections are collected to detect FITC or FITC-GCF at 1.0 h after injection. Sample fluorescent images show that FITC-GCF but not FITC are detected in the neurons of the ipsilateral brain in Sham animals. Both FITC and FITC-GCF are detected in the neurons of the ipsilateral brain in ischemic animals. FITC-GCF is colocalized with NeuN-positive neurons. Scale bar = 50 μm. **(B)** Summarized data indicate that the number of NeuN-positive neurons labeled with FITC-GCF is remarkably higher than that labeled with FITC in the ischemic brain. The number of NeuN-positive neurons labeled with FITC-GCF is remarkably higher in the ischemic brain than in the Sham brain. (*n* = 7, *p < 0.05 vs. I/R + FITC or Sham + FITC-GCF; ANOVA test). I/R: ischemia-reperfusion. **(C)** l-histidine (10 mmol) and FITC-GCF (150 mg/kg) are intravenously injected in rats at 3.0 h following ischemia-reperfusion. The brain sections are collected to detect FITC-GCF at 1.0 h after intravenous injection. The summarized data indicate that the uptake of FITC-GCF is inhibited by l-Histidine (*n* = 6, *p < 0.05 vs. I/R; Student t-test). **(D)** Dose-response inhibition of FITC-GCF (150 mg/kg) uptake by l-Histidine in the ischemic brain. IV: intravenous injection; I/R: ischemia-reperfusion; l-histidine: L-His.

We further showed that few FITC-labeled cells were localized in the ipsilateral ischemia brain but not in the ipsilateral sham brain (Figure 4A). However, FITC-GCF-labelled cells were localized in both ipsilateral ischemic and the sham brain with more FITC-GCF-labelled cells in the ischemic brain (Figure 4A). We then examined the colocalization of FITC or FITC-GCF with NeuN (a mature neuronal marker) in Sham and I/R animals. Immunocytochemical staining showed that the number of NeuN-positive neurons labeled with FITC-GCF was higher than that labeled with FITC in the ischemic brain (Figure 4B). The number of NeuN-positive neurons labeled with FITC-GCF was higher than that of the Sham brain (Figure 4B). These results together indicate that GCF, but not FITC, efficiently passes through BBB and enters the damaged neurons after ischemia-reperfusion injury.

PhT1 is an oligopeptide transporter that is known to regulate the cellular uptake of di/tripeptides (Wang et al., 2010; Smith et al., 2013). It has been reported that PhT1 is abundantly expressed in the rodent brain (Yamashita et al., 1997; Shen et al., 2004; Smith et al., 2004). However, the function of PhT1 in the brain is largely unknown. To test whether PhT1 mediates GCF transport through BBB, brain uptakes of FITC-GCF were determined in the absence and presence of l-histidine, a specific inhibitor of PhT1. As shown in Figure 4C, in the presence of 10 mmol l-histidine, the uptake of FITC-GCF was reduced by 57%. Dose-response inhibitory analysis indicated that the uptake of FITC-GCF (150 mg/kg) was inhibited by l-histidine in the ischemic brain region with IC50 of 4.61 mmol (Figure 4D). These data suggest that the transport of GCF through BBB requires oligopeptide transporter PhT1.

Interestingly, we revealed that the intranasal administration of FITC-GCF led to the distribution of FITC-GCF in the damaged rat brain region after ischemia-reperfusion injury (Supplementary Figure S1). This result further confirmed the permeability of GCF to BBB and also provides a possibility for the future development of GCF as a pre-hospital oneself therapy immediately following stroke onset.

### Intravenous GCF injection leads to an increased level of glycine in the ischemic brain

**FIGURE 5:**
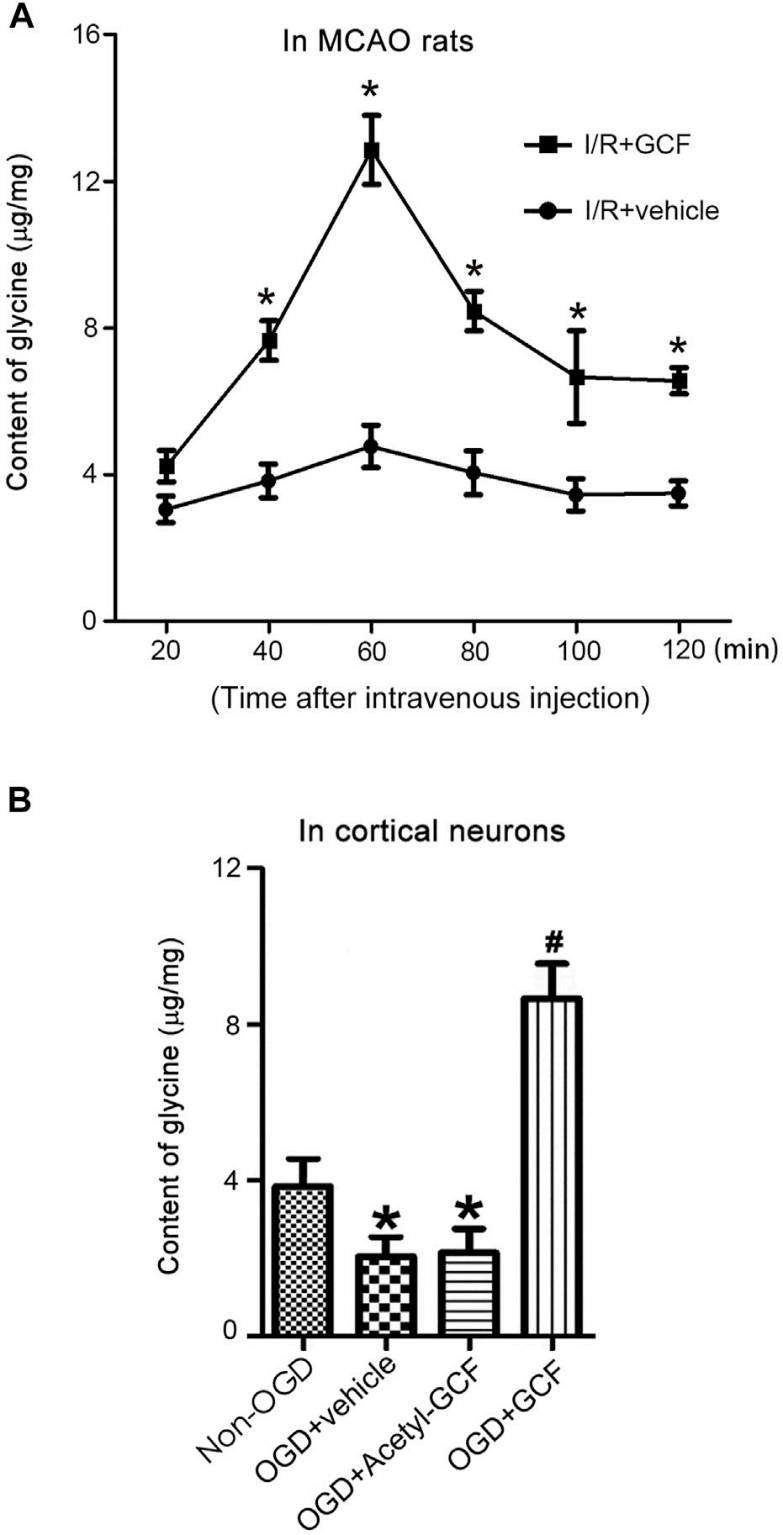
GCF injection results in increased level of glycine in the ischemic brain. **(A)** Intravenous injection of GCF (150 mg/kg) at 1.0 h after rat I/R increases the levels of glycine in the ischemic brain tissue at 40, 60, 80, 100 and 120 min following GCF injection (*n* = 6, *p < 0.05 vs. I/R + vehicle; ANOVA test). I/R: ischemia reperfusion; Vehicle: 0.9% NaCl. **(B)** HPLC measurement in the neuronal cultures show that GCF (15 μM) but not acetyl-GCF at 1.0 h after reoxygenation increases the level of intraneuronal glycine at 60 min following GCF treatment (n = 6, *p < 0.05 vs. Non-OGD, ^#^ *p* < 0.05 vs. OGD + vehicle; ANOVA test). Vehicle: 0.9% NaCl.

To determine whether the tripeptide GCF released glycine in the ischemic brain region, we performed HPLC to measure the levels of glycine in the ipsilateral ischemic brain tissues after intravenous GCF injection. Our results showed that the treatment of GCF (150 mg/kg) increased the level of glycine in the ischemic brain tissue at 40, 60 80, 100, and 120 min after GCF injection while compared with the vehicle group (Figure 5A). We also treated the neuronal cultures, subjected to oxygen-glucose deprivation (OGD, an *in vitro* model of cerebral ischemia injury), with GCF or acetyl-GCF [a stabilized form of GCF that was difficult to be cleaved (Xiong et al., 2009)] at 1.0 h after reoxygenation. HPLC was used to measure the level of intraneuronal glycine. We found that the level of glycine was increased at 60 min after the treatment of GCF but not acetyl-GCF (Figure 5B). These data support the possibility that GCF may release glycine in the ischemic brain.

### Intravenous GCF injection protects against cerebral ischemia-reperfusion injury

**FIGURE 6:**
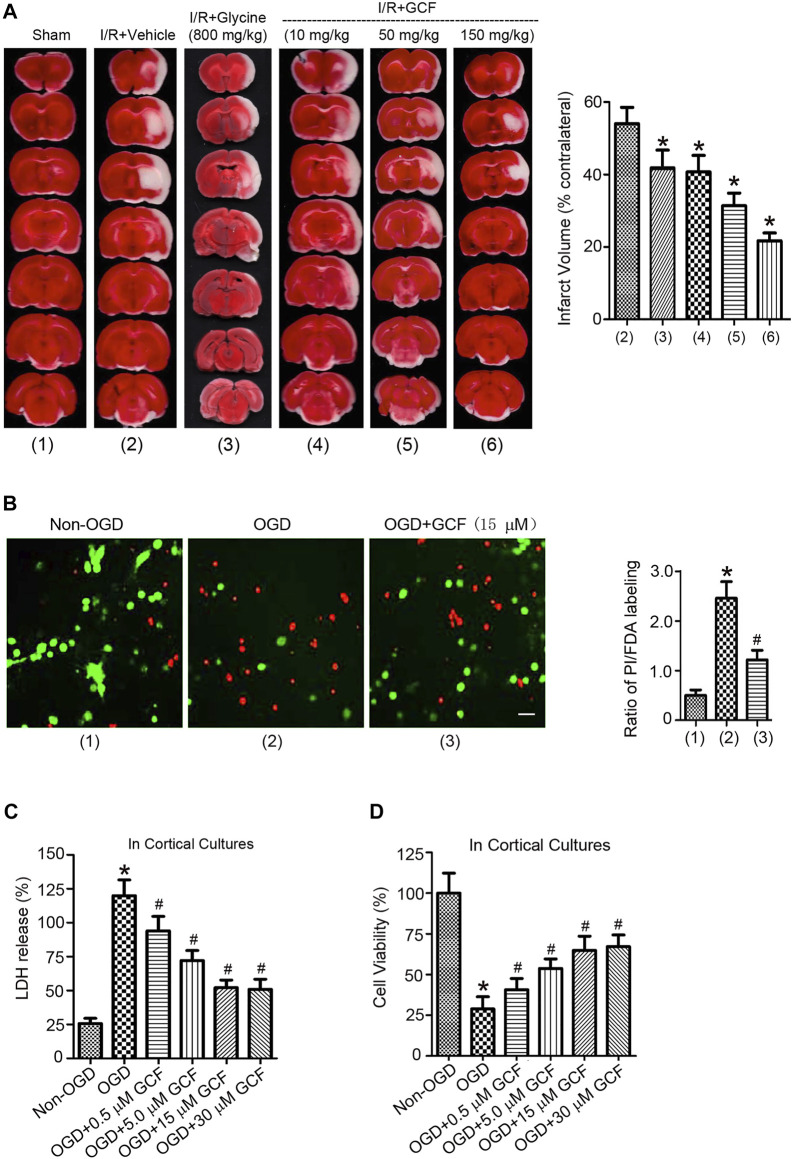
The neuroprotective effect of GCF. **(A)** Left: sample images of TTC staining show that intravenous injection of glycine (800 mg/kg) and GCF (10, 50 and 150 mg/kg) at 1.0 h following I/R reduces the infarct volume of ischemic brain at 24 h following ischemia onset. Right: the summarized data indicate that GCF is neuroprotective (*n* = 8, *p < 0.05 vs. I/R + Vehicle; ANOVA test). IV: intravenous injection. I/R: ischemia-reperfusion. Vehicle: 0.9% NaCl. **(B)** GCF (15 μM) reduces OGD-induced cortical neuronal death (n = 6, *p < 0.05 vs. Non-OGD; ^#^ *p* < 0.05 vs. OGD; ANOVA test). Green: FDA; Red: PI. **(C,D)** LDH and MTT assays show that GCF is neuroprotective in OGD-insulted neurons (*n* = 7 for each group, *p < 0.05 vs. Non-OGD; ^#^ *p* < 0.05 vs. OGD; ANOVA test).

We then set up to determine whether the intravenous injection of GCF was neuroprotective after ischemia-reperfusion injury. GCF was intravenously injected at 1.0 h after ischemia-reperfusion in rats subjected to 2 h MCAO. Animals were sacrificed to measure the infarct volume at 24 h after ischemia onset. The cerebral infarction was measured in the brain sections stained with TTC (2,3,5-triphenyltetrazolium chloride). Our data showed that intravenous injection of GCF (10, 50, and 150 mg/kg) reduced the infarct volume of MCAO rats (Figure 6A). Glycine (800 mg/kg) was also intravenously injected in the same experimental conditions. We found that an extra high concentration of glycine exerted a neuroprotective effect (Figure 6A).

We further verified the neuroprotective effect of GCF in the OGD-insulted cultured neurons. Double labeling of propidium iodide (PI) and fluorescein diacetate (FDA) was performed to determine the ratio of neuronal death/viability. We found that treatment of GCF (15 μM) at 1.0 h after reoxygenation increased the ratio of cortical neuronal viability at 24 h after OGD onset (Figure 6B). In a dose-dependent manner, GCF reduced the level of lactate dehydrogenase (LDH) released from the injured neurons and increased the rate of neuronal survival determined by 3-(4,5-dimethylthiazol-2-yl)-2,5-diphenyltetrazolium bromide (MTT) assay (Figures 6C,D).

### GCF promotes functional recovery of stroke animals

**FIGURE 7:**
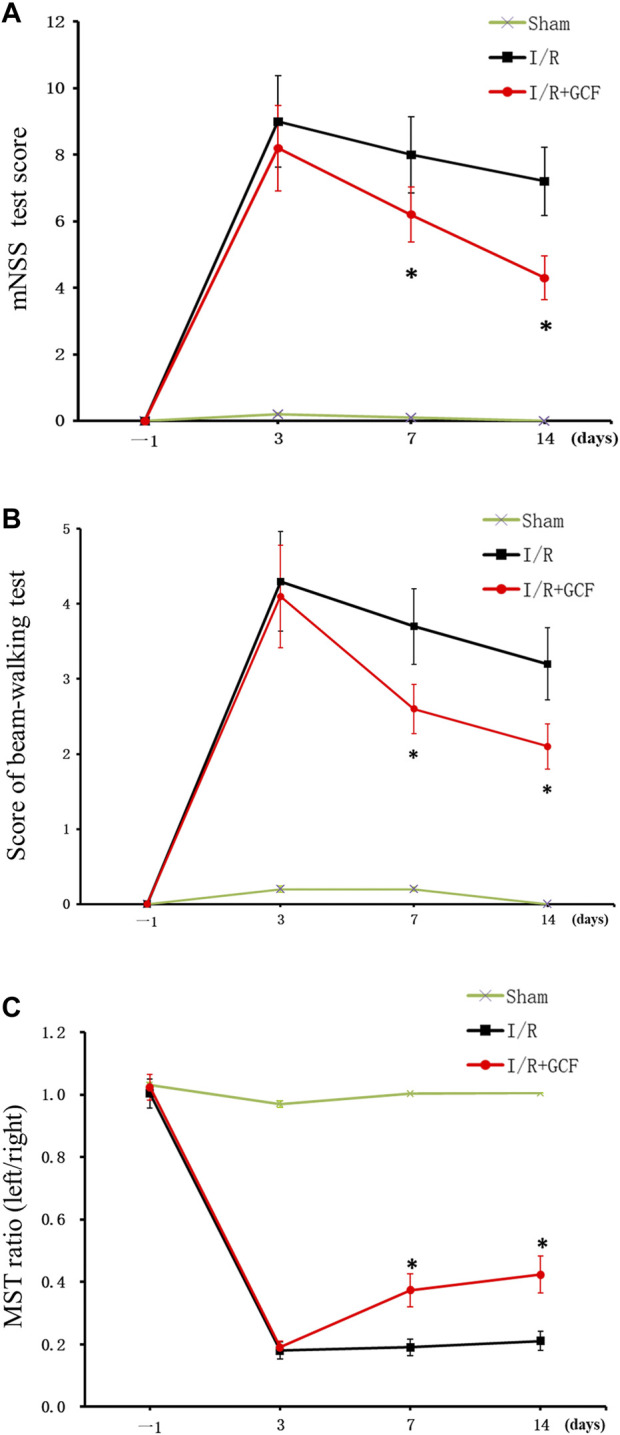
GCF Improves the neurobehavioral function of stroke animals. (A) Summarized data show that rats treated with GCF have significantly lower scores on mNSS tests on days 7 and 14 after MCAO (*n* = 8 rats for each group, *p < 0.05 vs. I/R, ANOVA test). (B) Summarized data show that rats treated with GCF have significantly lower scores on the beam-walking test on days 7 and 14 after MCAO (n = 9 rats for each group, *p < 0.05 vs. I/R, ANOVA test) (C) Summarized data show that rats treated with GCF have a significantly higher ratio of a modified sticky-tape test at day 7 and 14 after MCAO (*n* = 8 rats for each group, *p < 0.05 vs. I/R, ANOVA test).

To determine the functional consequence of GCF in stroke animals, we performed a battery of neurobehavioral tests including modified neurological severity scores (mNSS) test, beam-walking test, and modified sticky-tape (MST) test 1 day before MCAO, and at 3, 7, and 14 days after MCAO. Our data showed that rats treated with GCF had significantly lower scores on the mNSS test on day 7 and 14 after MCAO (Figure 7A), and lower scores on a beam-walking test on day 7 and 14 after MCAO (Figure 7B), and a higher ratio of modified sticky-tape (MST) test at days 7 and 14 after MCAO (Figure 7C). Together, these results provide functional evidence for the neuroprotective role of GCF in stroke animals.

### The neuroprotection of GCF depends on GCF-induced increase of glycine in the ischemic brain

**FIGURE 8:**
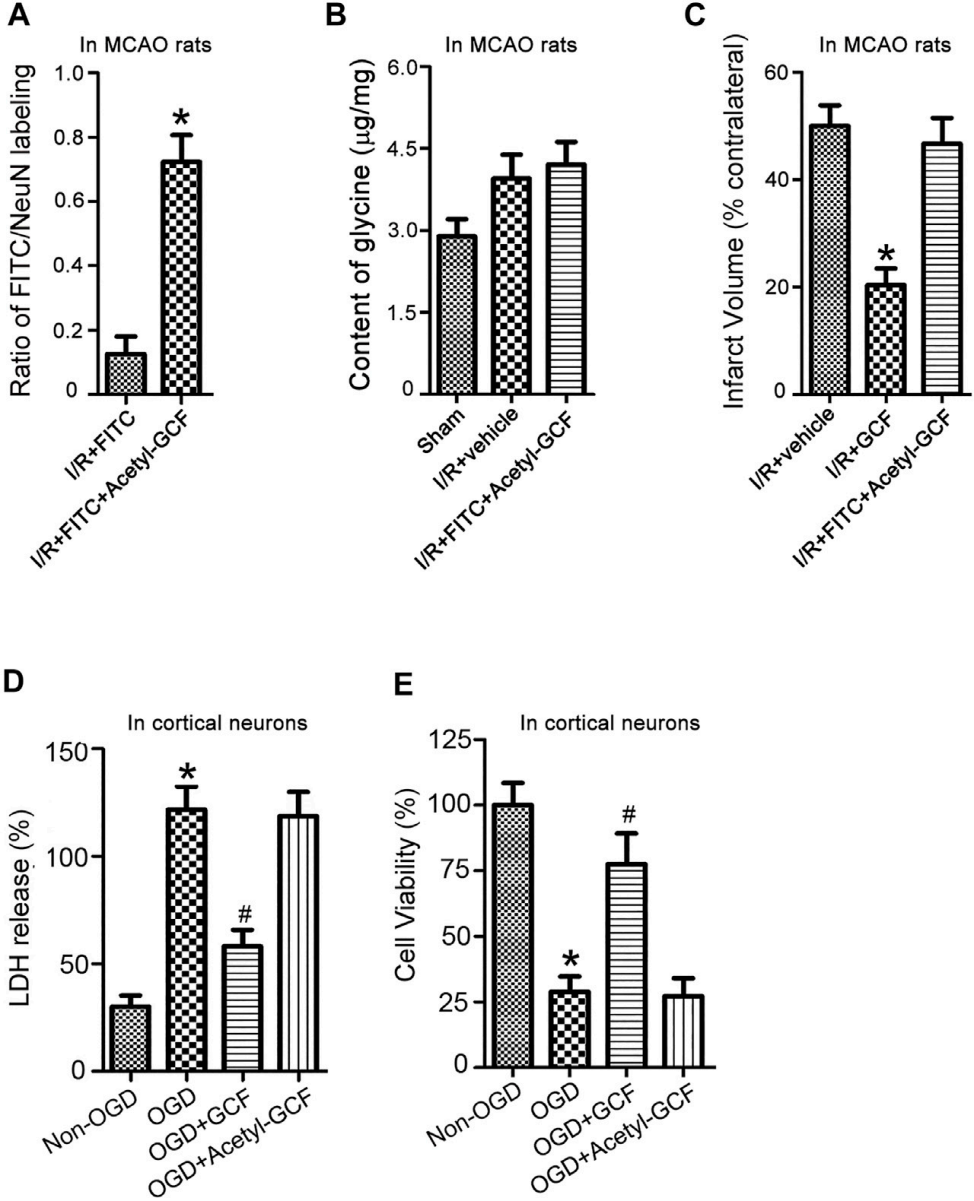
GCF-mediated increase of glycine in the ischemic brain is required for GCF-induced neuroprotection. **(A)** FITC-Acetyl-GCF (150 mg/kg) is intravenously injected in rats at 1.0 h following I/R. At 1.0 h after injection, FITC-Acetyl-GCF is localized in NeuN-labeled neurons (*n* = 7, *p < 0.05 vs. I/R + FITC; Student’s *t*-test). I/R: ischemia-reperfusion. **(B)** Compared with the group of I/R + Vehicle, the level of glycine is not increased in the ischemic brain at 1.0 h after the intravenous injection of Acetyl-GCF (150 mg/kg; n = 7). I/R: ischemia-reperfusion. **(C)** TTC staining data show that intravenous injection of Acetyl-GCF (150 mg/kg) at 1.0 h after I/R does not reduce the infarct volume at 24 h after ischemia onset (n = 7, *p < 0.05 vs. I/R + Vehicle; ANOVA test). I/R: ischemia-reperfusion. Vehicle: 0.9% NaCl. **(D,E)** LDH and MTT assays show that Acetyl-GCF (15 μM) does not reduce OGD-induced cortical neuronal death at 24 h after injury (*n* = 6 for each group, *p < 0.05 vs. Non-OGD; ^#^ *p* < 0.05 vs. OGD; ANOVA test).

The preceding HPLC data suggest that intravenous GCF injection led to an increase of glycine in the ischemic brain (Figure 5). To further verify whether the increased level of glycine contributed to GCF-induced neuroprotection, we synthesized a stabilized form of GCF, the Acetyl-GCF, which was difficult to cleave (Xiong et al., 2009). FITC-Acetyl-GCF (150 mg/kg) was intravenously injected at 1.0 h following ischemia-reperfusion. At 1.0 h after injection, FITC-Acetyl-GCF was found to localize in the ischemic neurons (Figure 8A). However, the level of glycine was not increased at 1.0 h after injection of Acetyl-GCF (Figure 8B). We also found that intravenous injection of Acetyl-GCF (150 mg/kg) 1.0 h after ischemia-reperfusion had no effect on the infarct volume at 24 h after ischemia onset (Figure 8C). In addition, Acetyl-GCF (15 μM) did not reduce OGD-induced cortical neuronal death (Figures 8D,E). Thus, these results suggest that intravenous injection of GCF confers neuroprotection by increasing the level of glycine in the ischemic brain region.

## Discussion

We and the others have previously shown that glycine exerts a neuroprotective effect *in vitro* and in cerebral ischemia injury *in vivo* (Yang et al., 2000; Zhao et al., 2005; Liu et al., 2007; Tanabe et al., 2010; Chen et al., 2016; Hu et al., 2016). However, whether these amino acids can efficiently cross the BBB to confer neuroprotection remains largely unknown. In this study we showed that a high dose of glycine was required for intravenous injection-induced neuroprotection in ischemic stroke rats, indicating a low BBB permeability of glycine in cerebral ischemia conditions. In order to deliver glycine through the BBB into the ischemic brain, we designed a novel glycine-containing and BBB-permeable tripeptide GCF. To ensure the tripeptide crosses the BBB, we included the aromatic amino acid phenylalanine in the tripeptide GCF. Indeed, our results showed that GCF was distributed in both Sham and ischemic brains but with a larger amount of GCF localized in the ischemic neurons following intravenous injection of GCF. These data support the notion that GCF can pass BBB efficiently. Since the GCF distribution is high in the ipsilateral brain compared with the contralateral side, it is expected that enhanced distribution of GCF is possibly due to BBB breakdown by ischemic damage or the induction of LAT1 expression at the BBB in the ischemic brain region.

Another key design for the GCF is the inclusion of cysteine with glycine to enable the release of glycine from GCF. It is possible that the specific peptidase expressed in neurons cleaves glycine in GCF. We showed that intravenous GCF injection led to an increased level of glycine in the ischemic brain and conferred neuroprotection. But the intravenous injection of acetyl-GCF, a non-cleaved form of GCF, resulted in no change in the level of glycine and exerted no neuroprotective effect on ischemic neuronal death. These data suggest that intravenous GCF injection-induced neuroprotection may be mediated by the released glycine from GCF in the ischemic brain.

Interestingly, intranasal administration of GCF also leads to the distribution of GCF in the ischemic brain region. This finding not only further supports the permeability of GCF to BBB but also provides an attractive possibility for the development of GCF as a pre-hospital neuroprotection therapy immediately after stroke onset.

Recent studies indicate that developing peptide-based neuroprotectants is a promising strategy in stroke therapy (Aarts et al., 2002; Cui et al., 2007; Hill et al., 2012; Hill et al., 2020). The peptide Tat-NR2B9c (also termed NA-1) is developed from the discovery that postsynaptic density protein-95, the NMDA receptor-interacting protein, is a hub for excitotoxic signaling and a stroke therapeutic target (Aarts et al., 2002; Cui et al., 2007). The Phase 3 clinical trial of NA1 has provided encouraging human efficacy data (Hill et al., 2020). In contrast to NA-1 and other existing peptides that use peptides themselves to interfere in signaling interaction or degrade target proteins (Aarts et al., 2002; Hill et al., 2012; Schaible et al., 2013; Fan et al., 2014), GCF acts as a BBB shuttle and prodrug to deliver its own amino acid to confer therapeutic effect, representing a new design of peptide drug and a novel therapeutic mechanism.

As a small peptide, GCF has a simple structure, low immunogenicity, and higher chemical versatility than traditional peptide approaches (Oller-Salvia et al., 2016). Tripeptide is easier to be characterized, synthesized, and purified and has a lower cost. Thus, the design of the BBB-permeable small peptide offers a safe, simple and efficient approach for the development of ischemic stroke therapeutics.

Substantial evidence indicates that amino acids, including glycine, have cytoprotective property (King et al., 2010; Petrat et al., 2012). Because of the low permeability of these amino acids to the BBB, amino acid supplement therapy for CNS diseases is lacking. The GCF design provides a novel therapeutic strategy by which low BBB-permeable amino acids are delivered into the brain of CNS diseases. In addition, the efficient membrane permeability of GCF also offers the possibility to develop GCF in treating a variety of periphery organ diseases including injuries to the liver, small intestine, lung, skeletal muscle, and heart following ischemia–reperfusion, hemorrhagic shock, or resuscitation (Petrat et al., 2012).

## Methods and materials

### Animals

All animal experiments were approved and carried out in compliance with the IACUC guidelines of the Wuhan University School of Medicine and Qingdao University School of Medicine. All animal use and experimental protocols were approved and carried out in compliance with the IACUC guidelines and the Animal Care and Ethics Committee of Wuhan University School of Medicine and Qingdao University School of Medicine. Randomization was used to assign samples to the experimental groups, and to collect and process data. The experiments were performed by investigators blinded to the groups for which each animal was assigned. All studies involving animals are reported in accordance with the ARRIVE guidelines for reporting experiments involving animals (Curtis et al., 2015; McGrath and Lilley, 2015).

### Reagents

GCF, Acetyl-GCF, FITC, FITC-GCF, FITC-Acetyl-GCF, and FITC-Glycine were obtained from Top-peptide Co., Ltd. (Shanghai, China). Glycine was purchased from Sigma-Aldrich (St. Louis, MO, United States). DNFB was purchased from Xiya Reagent (Chengdu, China). HPLC-grade acetonitrile and methanol were purchased from Sinopharm Chemical Reagent (Shanghai, China). All other chemicals of analytical reagent grade were purchased from Sinopharm Chemical Reagent (Shanghai, China).

### Middle cerebral artery occlusion in rats and infarct measurement

Adult male Sprague–Dawley (SD) rats, weighing 250–270 g, were group-housed with two to three rats per cage on a 12 h light/dark cycle in a temperature-controlled room (23–25°C) with free access to food and water. Animals were allowed at least 3 days to acclimatize before experimentation. Transient focal cerebral ischemia was induced using the suture occlusion technique (Wexler et al., 2002; Chen et al., 2017; Zhang et al., 2017). Male Sprague–Dawley rats weighing 250–270 g were anesthetized with 4% isoflurane in 70% N_2_O and 30% O_2_ using a mask. A midline incision was made in the neck, the right external carotid artery (ECA) was carefully exposed and dissected, and a 3–0 monofilament nylon suture was inserted from the ECA into the right internal carotid artery to occlude the origin of the right middle cerebral artery (MCA) (approximately 22 mm). After 1.5 h of occlusion, the suture was removed to allow reperfusion, the ECA was ligated, and the wound was closed. Non-OGD-operated rats underwent identical surgery except that the suture was inserted and withdrawn immediately. Rectal temperature was maintained at 37.0 ± 0.5°C using a heating pad and heating lamp.

Rats were sacrificed at various times following reperfusion after being anesthetized, and the brains were removed for TTC (2,3,5-triphenyltetrazolium chloride) staining. The brain was placed in a cooled matrix and 2.0 mm coronal sections were cut. Individual sections were placed in 10 cm Petri dishes and incubated for 30 min in a solution of 2% TTC in phosphate buffered saline at 37°C. The slices were fixed in 4% paraformaldehyde at 4°C. All image collection, processing, and analysis were performed in a blind manner. The scanned images were analyzed using image analysis software (Image-Pro Plus Version 6.0, United States). The infarct volume was calculated to correct for edema. The normal volume of the contralateral hemisphere and the normal volume of the ipsilateral hemisphere were measured, and the infarct percentage was calculated as % contralateral structure to avoid mismeasurement secondary to edema.

### Intravenous injection

Male Sprague-Dawley rats weighing 250–270 g were anesthetized with 4% isoflurane in 70% N_2_O and 30% O_2_ using a mask. FITC, FITC-GCF, GCF, and Acetyl-GCF at the designed concentration were dissolved in 0.5 ml saline and then injected into the right femoral vein per animal.

### Immunohistochemistry

The method has been described previously (Ning et al., 2004). Rats were sacrificed after being anesthetized, and the brains were removed. The tissue was then frozen in Tissue-Tek OCT mounting medium and 15 μm coronal sections were cut. Sections were subsequently spread on microscope slides and allowed to air dry. Air dried sections were fixed in 4% paraformaldehyde in PBS for 30 min then washed 3 times in PBS for 5 min each. After post-fixation, the sections were blocked and permeabilized in 0.1 M PBS with 0.3% TX-100 (sigma) and 5% bovine serum albumin (BSA) for 1 h. Following permeabilization, a primary anti-NeuN or anti-glycine antibody (Millipore, United States) was applied overnight at 4°C. Primary antibody was removed with three washes in PBS and secondary antibody (Alexa 594 conjugated to goat anti-mouse) was applied for 1 h at room temperature. Secondary antibody was removed with three washes in PBS and the sections were observed under an Olympus BX51 microscope or a Zeiss LSM 510 META confocal microscope.

### Neuronal culture and OGD insult

The cortical neuronal cultures were prepared from Sprague–Dawley rats at gestation day 17 as described (Brewer et al., 1993; Liu et al., 2006). Briefly, dissociated neurons were suspended in a plating medium (Neurobasal medium, 2% B-27 supplement, 0.5% FBS, 0.5 μM l-glutamine, and 25 μM glutamic acid) and plated on poly-d-lysine coated Petri dishes. After 1.0 days in culture, half of the plating medium was removed and replaced with a maintenance medium (Neurobasal medium, 2% B-27 supplement, and 0.5 mM l-glutamine). Thereafter, the maintenance medium was changed in the same manner every 3.0 days. To suppress glial cell growth, the cultures were exposed to 10 μM cytosine arabinoside (Ara C) for 48 h between days 3 and 5 after plating. The cultured neurons were used for experiments 12 days after plating.

The method of OGD insult was described in our previous study (Liu et al., 2006). To initiate the OGD challenge, cells were transferred to a deoxygenated glucose-free extracellular solution (ECS) (in mM: 116 NaCl, 5.4 KCl, 0.8 MgSO_4_, 1.0 NaH_2_PO_4_, 1.8 CaCl_2_, and 26 NaHCO_3_), introduced into a specialized chamber, and maintained at 37°C in 85% N_2_/10% H_2_/5% CO_2_ for 2 h. Neurons were removed from the chamber, transferred to the maintenance medium, and returned to the incubator. For non-OGD group treatment, cultures were transferred to the standard ECS (in mM: 116 NaCl, 5.4 KCl, 0.8 MgSO_4_, 1.0 NaH2PO_4_, 1.8 CaCl_2_, 26 NaHCO_3_, and 33 glucose), introduced into the chamber maintained at 37°C in 95% air/5% CO_2_. After 2 h incubation, the neurons were transferred to the maintenance medium and returned to the original incubator.

### High-performance liquid chromatography

The neuronal cultures were first washed with ECS three times before sample collection. Brain samples were collected after the animals were sacrificed and transcardially perfused with PBS. The samples were then homogenized in 200 μL of chilled saline solution (0.9%). The homogenate was centrifuged at 14,000 rpm for 20 min at 4°C. The supernatant was used to measure the level of proteins. The remaining samples were mixed with two x volume of methanol and centrifuged to remove protein. The supernatant was transferred for derivatization (Mao et al., 2012). An aliquot of mixed amino acids solution (40 μL) or sample supernatant (40 μL) was mixed with 0.5 M sodium bicarbonate solution (40 μL) and 0.5% of DNFB (20 μL) in a 500 μL centrifuge tube. The well-mixed solution was incubated at 60°C in the water bath for 1 h. DNFB was reacted with an amino group and enabled amino acids to be detected with UV detection. After cooling to room temperature, the solution (30 μL) was injected into the equilibrated HPLC system. Analysis was carried out on a Waters HPLC system (United States) equipped with a UV detector (360 nm). The analyte was separated on a Hypersil BDS C_18_ (200 mm length × 4.6 mm, 5.0 µm particle diameter) manufactured by Elite Analytical Instrument (Dalian, China). The mobile phase was composed of 0.05 M (PH = 6.5) sodium acetate buffer (A), a mixture of acetonitrile and water (1:1, v:v, B) was carried out in a gradient elution mode with a flow rate of 1.0 ml/min (Supplementary Table S1). The column temperature was maintained at 30°C (Zhang et al., 2012).

### PI and FDA labeling

Double staining of propidium iodide (PI) and fluorescein diacetate (FDA) was performed to detect neuronal death/viability (Jones and Senft, 1985; Zheng et al., 2012). Briefly, cultures were rinsed with an extracellular solution and incubated with FDA (5.0 μM) and PI (2.0 μM) for 30 min. The cultures were washed with ECS and then viewed on an Olympus fluorescent microscope (IX51, Olympus). Neuronal death/viability was determined by calculating the number of PI-labeled cells over FDA-labeled cells. The investigator for the cell count was blinded to the experimental treatment.

### LDH assay

The lactate dehydrogenase (LDH) is a cytoplasmic enzyme retained by viable cells with intact plasma membranes and released from cells with damaged membranes. The LDH release was measured using CytoTox 96 cytotoxicity kit based on the manufacturer’s instructions (Promega, United States) (Zheng et al., 2012). The levels of maximal LDH release were measured by treating the cultures with a 10× lysis solution (provided by the manufacturer) to yield complete lysis of the cells. Absorbance data were obtained using a 96-well plate reader (Molecular Devices, United States) at 490 nm. According to the manufacturer’s instructions, the LDH release (%) was calculated by calculating the ratio of experimental LDH release to maximal LDH release.

## MTT assay

The viability of the cells in the neuronal cultures was assessed by their ability to uptake thiazolyl blue tetrazolium bromide (MTT) (Chang et al., 2010; Chien et al., 2015; Chen et al., 2016). The cells were incubated with MTT for 1.0 h, then lysed with dimethyl sulfoxide (DMSO) and left at room temperature in the dark overnight. The lysates were then read on a plate reader (PowerWave X, Bio-Tek) at the absorbance wavelength of 540 nm.

### Neurobehavioral tests

The modified neurological severity score (mNSS) test for evaluating neurological function (Panahpour et al., 2019), the Beam walk test for measuring animals’ complex neuromotor function (Petullo et al., 1999; Chen et al., 2001), and the Adhesive-removal test, a modified sticky-tape (MST) test, for evaluating forelimb function (Aronowski et al., 1996) were described in detail in our previous study (Chen et al., 2017; Zhang et al., 2017).

### Statistical analysis

The data and statistical analysis in this study comply with the recommendations on experimental design and analysis (Curtis et al., 2015). The group sizes per experiment were based on a power analysis (Charan and Kantharia, 2013). The criterion for significance (alpha) was set at 0.05. The power of 80% was considered to yield a statistically significant result. Student’s *t*-test or ANOVA test was used where appropriate to examine the statistical significance of the differences between groups of data. Bonferroni tests were used for *post hoc* comparisons when appropriate. All results are presented as mean ± SEM. Significance was placed at *p* < 0.05.

## Data Availability

The raw data supporting the conclusion of this article will be made available by the authors, without undue reservation.
