# Supplementary material for: A synthetic BBB-permeable tripeptide GCF confers neuroprotection by increasing glycine in the ischemic brain
Source: Front Pharmacol. 2022 Aug 15;13:950376. doi: 10.3389/fphar.2022.950376 (PMC9420865; doi:10.3389/fphar.2022.950376)
Supplement: Supplementary file 1 [file DataSheet1.doc]

**A synthetic BBB-permeable tripeptide GCF confers neuroprotection by increasing glycine in the ischemic brain**

Juan Chen^1,2^*, Yang Zhuang^2^*, Ya Zhang^2^, Huabao Liao^2^, Rui Liu^2^, Jing Cheng^2^, Zhifeng Zhang^2^, Jiangdong Sun^3^, Jingchen Gao^3^, Xiyuran Wang^3^, Shujun Chen^3^, Liang Zhang^4^, Fengyuan Che^5^, Qi Wan^3,6^

^1^Department of Neurology, the Central Hospital of Wuhan, Tongji Medical College, Huazhong University of Science & Technology, 26 Shengli Street, Wuhan 430013, China

^2^Department of Physiology, School of Medicine, Wuhan University, 185 Donghu Street, Wuhan 430071, China

^3^Institute of Neuroregeneration & Neurorehabilitation, Department of Pathophysiology, School of Basic Medicine, Qingdao University, 308 Ningxia Street, Qingdao 266071, China

^4^Krembil Research Institute, University Health Network, University of Toronto, 399 Bathurst Street, Toronto, ON, Canada M5T 2S8

^5^Central Laboratory, Department of Neurology, Linyi People's Hospital, Qingdao University, 27 East Jiefang Road, Linyi, Shandong, China

^6^Qingdao Gui-Hong Intelligent Medical Technology Co. Ltd, 7 Fenglong Road, Qingdao High-tech Industrial Development District, Qingdao, China

*These authors contributed equally to this work.

Correspondence:

Professor Qi Wan, Institute of Neuroregeneration & Neurorehabilitation, Department of Pathophysiology, Qingdao University, 308 Ningxia Street, Qingdao 266071, China.

Email: qiwan1@hotmail.com

Dr. Fengyuan Che, Central Laboratory, Department of Neurology, Linyi People's Hospital, Qingdao University, 27 East Jiefang Road, Linyi, Shandong, China.

Email: che1971@126.com

Running title: A glycine-containing tripeptide confers neuroprotection


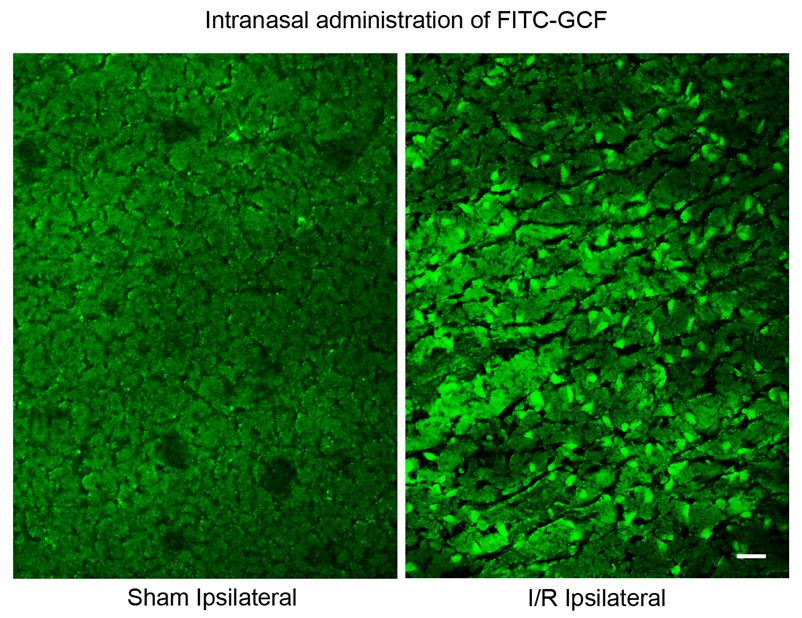


**Supplementary Figure 1. Intranasal administration of FITC-GCF leads to the distribution of FITC-GCF in the ischemic brain region after ischemia-reperfusion injury.** FITC-GCF (150 mg/kg) is intranasally applied at 1 h after I/R injury. The imaging shows the expression of FITC-GCF in the ischemic striatum at 1.0 h after FITC-GCF application. I/R: ischemia reperfusion.

**Supplementary Table 1** Gradient elution program used for the separation of amino acids derivatives under HPLC conditions.

| Time (min) | Eluent A (%) | Eluent B (%) |
| --- | --- | --- |
| 0 | 84 | 16 |
| 10 | 55 | 45 |
| 25 | 15 | 85 |
| 30 | 84 | 16 |
